# Designing Lifestyle Interventions for Common Mental Disorders: What Can We Learn from Diabetes Prevention Programs?

**DOI:** 10.3390/nu13113766

**Published:** 2021-10-25

**Authors:** Rachelle S Opie, Felice N Jacka, Wolfgang Marx, Tetyana Rocks, Claire Young, Adrienne O’Neil

**Affiliations:** Food and Mood Centre, IMPACT—The Institute for Mental and Physical Health and Clinical Translation, School of Medicine, Barwon Health, Deakin University, Geelong 3220, Australia; f.jacka@deakin.edu.au (F.N.J.); wolf.marx@deakin.edu.au (W.M.); tetyana.rocks@deakin.edu.au (T.R.); claire.y@deakin.edu.au (C.Y.); adrienne.oneil@deakin.edu.au (A.O.)

**Keywords:** common mental disorders, depression, anxiety, diabetes prevention programs, Type 2 Diabetes, lifestyle interventions

## Abstract

Lifestyle factors including diet, sleep, physical activity, and substance use cessation, are recognised as treatment targets for common mental disorders (CMDs). As the field of lifestyle-based mental health care evolves towards effectiveness trials and real-world translation, it is timely to consider how such innovations can be integrated into clinical practice. This paper discusses the utility and scale-up of lifestyle interventions for CMDs and draws on diabetes prevention literature to identify enablers and barriers to translation efforts. We discuss the extent to which lifestyle interventions aimed at managing CMDs and preventing diabetes share commonalities (program content, theoretical underpinnings, program structures, interventionists, frameworks promoting fidelity, quality, sustainability). Specific considerations when utilising these programs for mental health include personalising content with respect to symptoms and trajectories of depression and anxiety, medication regimen and genetic risk profile. As this field moves from efficacy to effectiveness and implementation, it is important to ensure issues in implementation science, including “voltage drop”, “program drift”, logistics, funding, and resourcing, are in line with evidence-based models that are effective in research settings. Ongoing considerations includes who is best placed to deliver this care and the need for models to support implementation including long-term financing, workforce training, supervision, stakeholder and organisational support.

## 1. Introduction

The common mental disorders (CMDs), depression and anxiety, are the leading contributors to the global burden of disability [1]. While the last decade has seen substantial efforts to reduce stigma and increase access to treatments, the population burden of depression and anxiety has not reduced and may in fact be increasing [2], especially in the COVID-19 setting [3]. This burden is exacerbated by the fact that mental disorders increase the risk of chronic physical disorders, such as cardiovascular disease, obesity, and diabetes, which are not only highly comorbid with mental disorders but also mutually reinforcing [4]. The past decade has seen the emergence of compelling top tier meta-analytic level data for the use of lifestyle-based programs for management of mental disorders [5,6]. These are predominantly in the areas of diet quality, physical activity, substance use cessation (drug, alcohol, smoking) and sleep quality [5].

These advances in evidence are very recently being reflected in policy and clinical practice guidelines, particularly in Australia where the recent Productivity Commission into Mental Health Report for the first time explicitly identified lifestyle behaviours, including diet, as both risk factors and treatment targets for mental disorders [7]. Concordantly, for the first time, the 2020 Royal Australian New Zealand College of Psychiatrists, in its updated clinical practice guidelines, stated that lifestyle approaches should form the foundation of treatment for mood disorders [8]. These very recent recommendations are now considered “non-negotiable and to be discussed with all patients” [8]. Similarly, guidelines by the European Psychiatric Association support the use of physical activity interventions in mild-moderate depression [9]. Given the recent advances in evidence supporting the efficacy of lifestyle interventions to manage mood disorders, as well as the clear imperative to address the very substantial burden of chronic non-communicable diseases—reflected in the large mortality gap—it is likely that such clinical guidelines will become more common. However, in order to move from clinical recommendations to full application and integration in mental health care, a clear understanding of how to best proceed in translating current knowledge into clinical care and public health policy is urgently needed. Looking to programs with the most established evidence in the field of *physical* health care, where lifestyle interventions have been a core component of interventions for decades, is likely to offer important insights into the most efficient and effective way of integrating lifestyle interventions into *mental* health care.

As part of the 2019 Lancet Psychiatry Commission of physical health in people with mental illness [10], the authors recommend the seminal diabetes prevention projects (DPPs) as an intervention model due to its major success at the population level in reducing diabetes incidence in high risk groups [10]. Indeed, this program has an extensive evidence base for efficacy and effectiveness in chronic disease management, it is the most commonly delivered lifestyle intervention in the community setting to individuals with elevated risk of T2DM, and has been successfully deployed and evaluated across numerous countries in both controlled and real-word settings [11,12,13,14,15]. As such, this paper aims to (i) summarise the current evidence base for the application of lifestyle programs in mental health treatment, (ii) provide a brief overview of the evolution of diabetes prevention programs, (iii) identify commonalities and differences for application in each program, (iv) ascertain what did and what did not work in the scale up of lifestyle-based diabetes prevention programs, and (v) discuss how these key concepts are central to translation efforts for the future of lifestyle-based mental health care. These understandings are critical if lifestyle approaches are to form the foundation of mood disorder treatment [5,6,16,17,18,19,20,21].

### 1.1. Evidence Base for the Use of Lifestyle Programs in Mental Health

*Diet quality:* Diet quality has consistently been shown to be independently related to depression prevalence and risk across countries and cultures [16]. More recently, randomised controlled trials (RCTs) have demonstrated the efficacy of targeting diet quality in substantially reducing symptoms of even severe depressive illness. A key example is the SMILES trial, a 12-week whole-of-diet intervention in 67 participants with moderate to severe depression that yielded significantly larger effects on depression (*Cohen’s d* = −1.16, *p* < 0.001) and anxiety scores (*Cohen’s d* = −0.594, *p* = 0.033) compared to the control condition (33% vs. 8% remission) [22,23]. Critically, program completion was high and the degree of symptom reduction was directly associated with adherence to the Mediterranean-style diet (“ModiMed diet”) [24]. This study also showed a cost savings of ~$2500 per participant in favour of the dietary intervention due to reduced healthcare costs and reduced productivity costs (e.g., missed fewer paid and unpaid work days) [23]. A larger, group-based study using a similar approach reported very comparable findings and was also highly cost-effective [25]. In addition, these studies are now corroborated by meta-analytic evidence, including data from 16 RCTs and 45,826 participants, showing that dietary improvement is also an efficacious treatment option for sub threshold depression [6], especially when delivered by accredited nutritionists or dietitians.

*Physical activity:* Physical activity is also a well-established risk factor for mental disorders [5,19]. Meta-analyses further show comparable effect sizes to that of dietary change for exercise interventions (*effect size Cohen’s d* = 1.42) [19,26]. These effects hold across ages, genders, ethnicities and socioeconomic status. The largest and most recent meta-analysis of 35 RCTs found medium effect size (but high heterogeneity) for adults with depressive disorders (Standardised Mean Difference (SMD) = −0.66, 95% Confidence Internal (CIs): −0.86 to −0.46). The evidence is relatively consistent that structured moderate-to-vigorous intensity exercise can have a positive effect on symptoms of depression as an add-on treatment to usual care in adolescents, working age and older adults [19]. Additionally, some evidence shows that muscular strength and resistance training also are protective against mental disorders [5]. In particular, strength training can significantly improve mental health, with effects that may persist over and above those of aerobic exercise alone [5].

*Sleep quality:* Individuals with anxiety and mood disorders often experience sleep architecture abnormalities including sleep continuing disturbances, prolonged sleep latency, decreased sleep efficiency, early morning awakenings, and decreased total sleep time [27]. Yet, the relationship between sleep and mental disorders appears to be bidirectional in that sleep and insomnia are also an important predictor of risk for mental health problems [5]. For example, a study of community-based adolescents showed at least 3 x greater likelihood of anxiety disorders and major depression among individuals with insomnia compared to those without [28]. Moreover, there is a significant body of evidence that poor sleep is a key modifiable lifestyle factor, with findings showing that poor sleep plays an important role in the onset and exacerbation of mental disorders. A 2019 meta-analysis of seven RCTs evaluating non-pharmacological sleep interventions found a large, significant reduction in depressive symptoms owing to treatment compared to controls (SMD = 0.81, 95% CI: 0.49–1.13, *I*^2^ = 27%) [5].

*Substance use reduction (tobacco, drug, alcohol):* There are fewer efficacy data for substance use cessation; however, meta-analytic evidence of smoking cessation from prospective studies show positive associations between quitting compared with continuing smokers for anxiety (standardised mean difference: −0.37 (95% CIs: −0.70 to −0.03) and depression (SMD: −0.25 (−0.37 to −0.12) [20]. These results have been corroborated by some, but not all, recent Mendelian randomization studies [29,30,31]. Furthermore, a recent Cochrane review of 102 smoking cessation intervention studies reported that smoking cessation interventions provided small to moderate improvements in mental health [32]. Meta-analytic evidence shows that substance use interventions also produce mental health improvements [21]. Recently, an online program targeting substance use, depression, and anxiety in adolescence from 88 schools decreased the odds of drinking, and reduced anxiety symptoms over a 30-month period [33].

*Multi-component lifestyle interventions:* When combined, the effects of lifestyle-based mental health care that addresses all targets, are likely enhanced and may optimise the effects of current first-line treatments. An adjunctive lifestyle-based model of care that drew on cognitive behavioural therapy and behavioural activation for patients with depression after a cardiac event reduced depression compared to a usual care control and its effects were far more pronounced for those with a lifetime history of major depression (*effect size Cohen’s d* = 0.3 *versus* 0.6) [34]. Furthermore, a recent meta-analysis of 50 RCTs found that multi-component lifestyle interventions reduced depressive symptoms compared to usual care [35].

### 1.2. Evidence Base for the Use of Lifestyle Programs in T2DM

DPPs are an effective way of preventing the onset of T2DM in those with pre-diabetes [36]. These lifestyle interventions are most commonly delivered in the community setting to individuals identified as being at elevated risk of developing T2DM. They have been successfully deployed and evaluated across Finland, China, Australia, the United States, the United Kingdom, and other countries in both controlled and real-world settings [11,12,13,14,15,37]. DPP lifestyle interventions have used an individual and/or group-based approaches, with varying session intensity (e.g., seven sessions during the first year and one session every three months thereafter [12], 16 sessions over 24-weeks [14], and six sessions over approximately eight months [13]). Key components of DPP lifestyle interventions include improving participant knowledge of T2DM, nutrition and physical activity; behavioural self-management, self-efficacy and self-monitoring of nutrition and physical activity; problem-solving and goal setting; social support; feedback (e.g., BMI, blood pressure, cholesterol); adoption and maintenance (e.g., addressing motivational challenges for sustaining lifestyle changes). Delivered by trained facilitators (background in psychology, exercise physiology, nutrition/dietetics, or nursing), DPPs tend to focus on five lifestyle change program objectives for participants [14]: (1) No more than 30% of daily energy from fat; (2) No more than 10% of daily energy from saturated fat; (3) At least 15 g/1000 kcal of fibre daily; (4) At least 30 min per day of moderate intensity physical activity; and (5) At least 5% weight loss.

The Finnish Diabetes Prevention Study was the first efficacy trial to demonstrate that an integrated lifestyle intervention could decrease diabetes incidence in at risk individuals by 58% over 3.2 years [12]. These effects were sustained nine years after intervention cessation [19,38]. Real world implementation and effectiveness was subsequently shown as part of the Good Ageing in Lahti Region (GOAL) program—a lifestyle implementation trial designed for the primary health care setting, with lifestyle and risk reduction objectives derived from the Finnish Diabetes Prevention Study [39]. The Finnish National Diabetes Prevention Program (FIN-D2D) then expanded its coverage nationally, and was the first national effort to implement prevention of diabetes in a primary health care setting, covering a population of 1.5 million [40]. In Australia, the DPP lifestyle intervention was adapted from the Finnish setting and delivered in a group-based setting to those at high risk of developing T2DM [41]. Early adaptation of the program significantly reduced diabetes risk, reduced body weight and waist circumference, and improved cardiovascular risk profile (e.g., serum lipids, plasma glucose and blood pressure) at 1 year follow-up [41,42]. The Life! Program, based on this intervention model, was then rolled out across Victoria in South Eastern Australia where it is now delivered to ~6000 individuals annually on behalf of its Government Department of Health and Human Services by peak consumer body Diabetes Victoria. Life! participants have achieved multiple positive outcomes including improved diet quality, increased physical activity and reduced sedentary behaviour, decreased weight and waist circumference, and reduced blood pressure [43]. Life! is one of the largest real-world implementation of a lifestyle program globally; details of its scale up have been described previously [44]. The US National DPP [37] and the English National Health Service (NHS) DPP [11] offer other examples of the largest available programs.

Additionally, DPPs offer sound return on investment (ROI). When delivered in the UK, the 20-year ROI for the National Health Service (NHS) DPP was £1.28 (AUD$2.38) for every £1 (AUD$1.86) invested [45]. With more than two decades of experience in the delivery and evaluation of DPPs throughout the world and more than a decade of insights from the more recent field of lifestyle psychiatry, there are several notable lessons that can be learnt from this program approach to produce better mental and physical health outcomes in those living with a CMD.

### 1.3. Can the Lessons Learnt from the DPP Help Us Better Tailor Lifestyle Psychiatry Programs?

It stands to reason that the application of DPP-style lifestyle interventions are likely to be effective in preventing physical health complications including cardiovascular disease and diabetes in individuals with CMDs. This is because CMDs and T2DM (i) share risk pathways, (ii) frequently co-exist and (iii) may share lifestyle targets for their self-management and prevention. Indeed, a DPP conducted in the Australian primary health care, delivered in a group-based setting, showed that it not only significantly reduced risk factors for T2DM over 12 months but also improved psychological measures (e.g., distress, depression, and vitality) [41]. The authors of the Lancet Psychiatry Commission highlight the need for replication of this program as a transdiagnostic lifestyle intervention for people with mental illness. But how might we adapt DPP style programs for the management of CMDs? We need to consider: (a) program content (suitability of goals to mental health populations); (b) personalisation based on risk profile (genetic, biological, social); (c) recruitment and retention; (d) effect maintenance (long term behaviour change in this population); and (e) future scalability (program fidelity, evaluation, quality indicators). Refer to Table 1 for a summary of the key considerations for each program domain.

### 1.4. What Factors Should Be Considered for Adaptation and Future Scale Up in Lifestyle Psychiatry?

Over the past 15 years in T2DM prevention, we have learnt that the effects of complex, lifestyle-based programs are often diluted when moving from controlled to real world settings [13]. This potential for reduced effects is referred to as “voltage drop” or a “scale-up penalty”, and needs to be considered as we move from efficacy trials to effectiveness and implementation trials in lifestyle psychiatry. Many interventions, as they were originally developed and tested, are not suitable for delivery at scale and need considerable adaptation to enable population-wide implementation [46]. For example, a recent systematic review of obesity interventions (including physical activity and nutrition outcomes) found that scaled-up interventions typically represented less than 75% of the effect established in efficacy trials of the intervention [46]. Additionally, a study that evaluated primary care physician interactions with their patients found that very little time was spent in lifestyle discussions. On average, physical activity was discussed for <1 min and nutrition for <90 s, with only approximately 10% of the encounter spent on physical activity, nutrition, or smoking topics. Whereas lifestyle discussions were more likely to occur during longer visits [47]. Indeed, there are critical elements that can promote or impede their widespread real-world implementation and scaling-up, including clinicians’ time restraints, competing demands, lack of adequately trained and skilled workforce, and financial reimbursement models. Considering the following may help mitigate some such issues.

*(a) Program content*: First, we need to consider if the original DPP lifestyle intervention objectives designed for those at risk of T2DM are appropriate for those with CMDs. Upon considering the extent to which the evidence from lifestyle psychiatry supports the use of the existing five DPP goals, the one-size-fits-all approach is likely unsuitable for those with an established CMD. For example, Goal 5, “at least 5% weight loss”, may not be appropriate for several reasons. The extensive epidemiological evidence linking diet quality to depression prevalence and risk consistently demonstrates associations that are independent of body weight [48]. Moreover, dietary interventions are efficacious in improving clinical depression in the absence of weight loss. For example, the average BMI of participants in the SMILES trial was 30 kg/m^2^ and this was unchanged at the end of the study, yet this intervention resulted in profound mental health improvements [22]. The pressure to lose weight can also add another mental health burden and challenge [49]. Shifting the emphasis from a weight-focus to a more health-focused paradigm is also more consistent with the relatively new Health at Every Size (HAES) approach. HAES aims to move away from weight-based discourse and is a promising public health response to obesity and other chronic diseases. Importantly, HAES addresses weight bias and stigma and has been shown to be effective in reducing psychological distress [49]. It may however still be worth exploring the associations of waist circumference or waist-to-hip ratio and depressive symptoms, independent of BMI or weight changes [50]. Especially considering the strong association between abdominal (visceral) obesity, metabolic syndrome, T2DM and cardiovascular disease [51].

Adapting Goals 1–4 in a way that integrates the most up-to-date evidence from lifestyle psychiatry is also critical. For example, Goal 1, “no more than 30% of total energy from fat”, is incompatible with adherence to a Mediterranean-style diet, rich in olive oil, nuts and seeds, and fatty fish, with an emphasis on the importance of omega-3 fatty acids, which can increase adult hippocampal neurogenesis (AHN) and decrease depressive symptoms [52]. Given smoking, alcohol and drug use is prevalent in individuals with CMDs, there may be a need for flexibility around the nomination of additional lifestyle targets that are critical to achievement of the core goals. These may include reducing use of alcohol, smoking, and/or drug use, and/or improved sleep hygiene. Although, it is worth noting that the meta-review evidence of interventions targeting these lifestyle pillars is stronger for the treatment of mental disorders than the prevention data [5]. It is important to consider the extent to which these lifestyle factors interact and can either accentuate or retract from the effects or increase the burden relating to too many change targets. Ultimately, evidence from the field of lifestyle psychiatry should be incorporated into program delivery by updating current goals and making them relevant for individuals with CMDs and cardiometabolic disorders.

*(b) Personalisation*: Personalisation of interventions, that draw on information on individual characteristics (e.g., genetic, psychiatric, and social profile) to develop targeted advice, may be more effective in achieving sustained behaviour changes with subsequent improvements in health outcomes, as well as reducing adverse events [53]. Additionally, mental health care programs should also consider the symptoms and trajectories of depression and anxiety, and medication regimen. For example, addressing behaviours, preferences and barriers by utilising behaviour change techniques (including goal setting, problem solving, feedback and monitoring) is likely integral to program success [24,54]. Importantly, appropriately qualified facilitators (e.g., accredited exercise physiologists and accredited dietitians) with advanced training in motivational interviewing, lifestyle counselling, and communication skills is necessary for implementing such programs [54,55].

Predictors of response sub-studies of Finnish Diabetes Prevention Study participants provide key insights into who may or may not benefit from a DPP lifestyle intervention. Which, given the shared pathways between diabetes and CMDs, are of interest in lifestyle psychiatry. For example, serum indolepropionic acid (IPA) at baseline has been shown to be associated with a reduced likelihood of developing T2DM [56]. IPA concentrations were also found to be directly associated with insulin secretion [57] which is of relevance in psychiatry, given that IPA has also been associated with antioxidant and anti-inflammatory effects in animal models, consistent with the proposed role of inflammation as a mutual risk factor for diabetes and depression. Research that has investigated biological predictors of treatment response to lifestyle intervention in psychiatry is an emerging area and currently, there is no primary candidate marker. The use of ‘omics’ sequencing, advanced analysis such as machine learning and emerging implicated pathways such as the gut microbiome may provide new avenues for identifying such markers [58].

*(c) Recruitment and retention*: Recruitment and retention challenges need to be addressed, especially considering that real-world diabetes prevention programs have experienced difficulties. The SMILES trial provides potential insights in a population with CMDs. Fifty-six individuals (83.6%) completed the assessment at the 12-week endpoint, with significantly more completers in the dietary intervention group (93.9%, *n* = 31) than the social support control group (73.5%, *n* = 25), χ^2^ (1) = 5.08, *p* = 0.024. Importantly, the high completion rates in the intervention group point to the acceptability of the dietary intervention to participants [22]. It is also worth drawing on learnings of other typically ‘hard to reach populations’ to provide insights. The MAGDA-DPP study was a RCT to assess the effectiveness of a structured DPP for women with previous Gestational Diabetes Mellitus (GDM), and provides insights into the potential retention rates of women in the post-partum period. Notwithstanding that postpartum depression has a high prevalence and poses a major public health challenge [59], this study achieved good retention rates (93% for the usual care group and 85% for the intervention group, when pregnancy was removed from loss to follow-up data) [60]. However, it is acknowledged that participation rates were low—the prominent symptoms of CMDs, such as fatigue and lack of motivation, and a requirement to attend a study centre on several occasions likely poses challenges. This indicates a potential need to utilise different methods for delivering interventions that do not require in-person attendance, such as telephone or web-based interventions. Importantly, programs need to invest in designing recruitment strategies that ensure high program reach and explore factors that optimize engagement and retention. We have shown that when delivered to individuals with depression or low mood after a cardiac event, not only is a telephone delivered model of care targeting lifestyle change feasible and effective (“MoodCare trial” effect size 0.30–0.60, *n* = 121) but has low attrition and high engagement over 6-months (median = 8/10 sessions, total 384 min for each participant) [34]. Moreover, effects can be maintained 6 months after cessation [61].

It is worth noting that there has been a significant increase in the prevalence of both major depressive disorder (with an estimated additional 53.2 million cases worldwide) and anxiety disorders (76.2 million additional cases) since before the pandemic [62].This means there will likely be more demand and uptake of such programs. We have observed this as part of our CALM trial (ACTRN12621000387820) which has partnered with a local tertiary mental health service to provide lifestyle based mental health care as part of a real-world trial. To date, we have recruited 273 adults with distress in 23 weeks and enrolled 41%.

*(d) Effect maintenance and program sustainability*: The original DPP incorporated “booster” sessions once participants had completed the original intervention period, and appeared potentially beneficial for supporting long term behaviour change [63]. Individuals experiencing depressive or anxious symptoms may require a different schedule of intervention that has consideration of their level of motivation and illness trajectory including remission prevention. Additionally, including peer support from individuals with lived experience, who act to enhance social support and self-efficacy, and promote problem-solving skills and knowledge development, may also offer an alternative method to achieving sustained lifestyle changes [64]. Meta-analytic evidence shows that peer support interventions help reduce symptoms of depression [65,66]. However, to ensure that peer workers can support lifestyle modification it would be beneficial for the workers to undertake health coach training. Moreover, research into whether peer support is a cost-effective approach that could be scaled-up for wider public health benefit is currently scarce.

Key factors for the successful scale up of DPP from efficacy trials to intervention programs were identified based on the theory of diffusion of innovation initially proposed by Rogers [67] and included the following: relative advantage over the status quo, compatibility with values and behaviours, lack of complexity, and trialability and observable results. Barriers included external project funding within a limited time frame, and preparation of counselling nurses. It was established that further training, support, and practical experience was required. The key factor of compatibility of values and behaviours identified in the Oldenburg review [44] refers to the general acceptability of diabetes as a public health burden, and the subsequent willingness of the population and key stakeholders to act in favour of diabetes prevention.

Despite increasing evidence for the immense public health burden of CMDs, there is wide variability in the way that mental illness and mental health is conceptualised by both key stakeholders and the general public, comparative to chronic physical ill-health [68]. Other considerations for program sustainability include the extent to which those most disadvantaged by socioeconomic or geographical characteristics can maintain momentum. Socioeconomic circumstances and context is critical for effective lifestyle based interventions especially when considering that mental disorders occur on a social gradient [69]. Identifying socioeconomic constraints around lifestyle modification, uptake, adherence and completion of health promotion interventions is critical. Social needs screening at the commencement of such programs can aid tailoring of program content and include tips and strategies for purchasing, preparing and storing nutrient dense meals under conditions of low resource or during periods when illness acuity is more pronounced. In the described above SMILES trial, participants typically consumed a diet that was more expensive than the prescribed ModiMed diet (Modified Mediterranean), which would achieve a savings of approximately AUD$25 per week ($138 and $112, respectively) [70]. Thus, considering affordability as part of the program development and delivery will be critical.

*(e) Evaluation, quality indicators*: Key differences exist between diagnosing T2DM and CMDs, an important consideration when measuring program success. For the original DPPs, the existence of diagnostic pathology measures (e.g., oral glucose tolerance test, fasting blood glucose, HbA1c) helps determine eligibility as well as primary outcomes [39]. In psychiatry, no such pathology testing exists. For decades, diagnostic biomarkers have evaded the field and as such, remains reliant upon patient self-report and clinician ratings and interviews. Embedding biomarker testing within DPP-style interventions can advance our understanding of risk assessment and treatment response and aid the development and validation of risk equations (e.g., The Australian Type 2 Diabetes Risk Assessment Tool, ‘AUSDRISK’), and if DPP style programs were initiated and funded en masse as part of standard population mental health, this could be a possible by-product of investing in this approach. The assessment tools should appropriately target populations with CMDs and allow for repeat measures.

Related to this issue of defining “success”, is defining quality to ensure program fidelity (e.g., that the program has been delivered as intended to promote replicability and scalability). As per our work in cardiac rehabilitation [71], developing quality assessment tools has been shown to mitigate the risk of care variation in care and “voltage drop” by maintaining treatment fidelity in the translation of complex interventions [13,72]. Greater attention needs to be paid to documenting intervention reach, adoption, implementation, and maintenance [72]. We have co-produced national quality indicators to guide everyday care practices of clinicians through standardisation and benchmarking [71,73]. The provision of these indicators allows for ongoing monitoring, benchmarking, evaluation and improvement of evidence-based clinical care, which is especially critical for lifestyle medicine given wide variation in delivery that can occur in their absence. It remains to be seen what are the most important quality indicators of care in lifestyle psychiatry; however, patient satisfaction, clinician rating, patient mental health rating, carer satisfaction, and health care costs may be considered. Participatory approaches including Delphi studies and field testing are required to achieve consensus from the various stakeholders (e.g., within mental health, public health and medical sectors) to ensure that we are meeting needs of patients, carers, clinicians and service providers, health administrators and policy makers when we consider reimbursement and funding models for this type of discrete health service. Co-design techniques that are now recognised as pivotal in the development, iteration and roll out of behavioural programs are especially important in mental health care given there has historically been significant neglect of the lived experience perspective.

## 2. Summary

In order for lifestyle-based mental health care to be integrated into practice in a manner comparable to physical disorders, the field now requires a concerted and coordinated effort to progress implementation, ensuring rigorous scientific research evidence and translational outcomes that can benefit the population. Importantly, there needs to be unified acceptance and understanding of CMDs by key stakeholders and the general public to achieve successful scale-up. The field of diabetes prevention from 20 years of successfully conducting complex, lifestyle-based programs in a way that provides sustained and cost-effective results, offers an attractive and relevant approach to the management of CMDs. Here, we have drawn on key lessons and principles learnt from the field of diabetes prevention, and have offered preliminary suggestions and recommendations critical in supporting the evolution of lifestyle psychiatry from efficacy to effectiveness and beyond. To translate such a model of lifestyle-based mental health care into routine practice in a way that meaningfully improves mental health outcomes of individuals at the population level, lifestyle programs should consider suitability of program content to mental health populations, personalisation based on risk profile, effect maintenance and program sustainability, and future scalability.

## Figures and Tables

**Table 1:** Summary of program domains and key considerations for adapting DPP style programs for the management of CMDs.

Program Domain	Key Considerations
Program content	Suitability of goals to mental health populations. Integrating the most up-to-date evidence from lifestyle psychiatry is critical. A one-size-fits-all approach is likely unsuitable. Flexibility around the nomination of additional lifestyle targets that are critical to achievement of the core goals.
b. Personalisation	Acknowledging the shared pathways between T2DM and CMDs, studies investigating the mechanisms underpinning treatment response in diabetes may provide key insights. Personalisation based on individual characteristics (e.g., psychiatric, genetic, biological, social profile) and environmental factors (e.g., settings, availability) may help maximise benefits as well as reduce adverse events. Behavioural change techniques should be utilised by appropriately qualified facilitators with advanced training in motivation interviewing, lifestyle counselling, and communication skills.
c. Recruitment and retention	Potential recruitment and retention difficulties need to be considered. Due to the prominent symptoms of CMDs, there is a potential need to utilise different methods for delivering the intervention that do not require in-person attendance, such as telehealth. Programs need to invest in designing recruitment strategies that ensure high program reach and explore factors that optimize engagement and retention.
d. Effect maintenance and program sustainability	Individuals with CMDs may require a different schedule of intervention that considers illness trajectory, including remission prevention. Peer support from individuals with lived experience may help achieve sustained lifestyle changes. Identifying socioeconomic constraints around lifestyle modification, uptake, adherence and completion of health promotion interventions is critical. For successful scale-up, require external project funding, and unified acceptance and understanding of CMDs by key stakeholders and the general public.
e. Evaluation, quality indicators	Embedding biomarker testing within lifestyle interventions can advance our understanding of risk assessment and treatment response and aid the development and validation of risk equations. Define quality indicators to ensure program fidelity. Also allows for ongoing monitoring, benchmarking, evaluation and improvement of evidence-based clinical care.
